# Effects of Combination Treatment with Leptin and Liraglutide on Glucose Metabolism in Insulin-Dependent Diabetic Mice

**DOI:** 10.3390/ijms26104595

**Published:** 2025-05-11

**Authors:** Linlin Fu, Mariko Sugiyama, Shahriar Kamal, Tsubasa Ide, Tadashi Takeda, Mitsuhiro Kuno, Hiroshi Takagi, Teruhiko Koike, Hiroshi Arima, Ryoichi Banno

**Affiliations:** 1 Research Center of Health, Physical Fitness and Sports, Nagoya University, Fro-cho, Chikusa-ku, Nagoya 464-8601, Aichi, Japan; 2 Department of Endocrinology and Diabetes, Graduate School of Medicine, Nagoya University, 65 Tsurumai-Cho, Showa-ku, Nagoya 466-8560, Aichi, Japan; 3 Department of Endocrinology and Diabetology, Nagoya City University East Medical Center, 1-2-23 Wakamizu, Chikusa-ku, Nagoya 464-8547, Aichi, Japan

**Keywords:** glucagon-like peptide-1 receptor agonist, liraglutide, leptin, IDDM, glucose metabolism

## Abstract

We investigated whether the peripheral co-administration of leptin and liraglutide (a glucagon-like peptide-1 receptor agonist) improved glucose metabolism in a mouse model of insulin-dependent diabetes mellitus (IDDM). Twelve-week-old male C57BL/6J mice were injected intraperitoneally with a high dose of streptozotocin to induce IDDM or vehicle-treated. Mice with IDDM were divided into four groups: leptin treatment alone (LEP), liraglutide treatment alone (LIRA), co-administration of leptin and liraglutide treatment (LEP+LIRA), untreated mice (UNT). Vehicle-treated mice were the healthy controls (HC). The blood glucose (BG) levels were measured, and a glucose tolerance test (GTT) was performed to compare the five groups. Leptin was administered peripherally at 20 μg/day using an osmotic pump, while liraglutide was administered subcutaneously at 1000 μg/kg/day. Monotherapy with leptin or liraglutide significantly improved glucose metabolism, as assessed by comparing BG levels and GTTs with those of the UNT group. Mice in the LEP+LIRA group showed even greater improvements in glucose metabolism than the monotherapy groups. Notably, glucose metabolism in the LEP+LIRA group improved comparably with the HC group. Thus, the peripheral co-administration of leptin and liraglutide effectively improved glucose metabolism in mice with IDDM without the use of insulin.

## 1. Introduction

Type 1 diabetes mellitus (T1DM) is characterized by hyperglycemia due to an insulin deficiency resulting from the destruction of pancreatic β-cells [1]. Insulin-dependent diabetes mellitus (IDDM) is a common condition observed in T1DM, for which insulin administration is the standard treatment for managing glucose metabolism abnormalities [2]. However, insulin therapy is associated with various adverse effects, including severe hypoglycemia [3] and ectopic fat accumulation, which can lead to atherosclerosis [4,5]. Additionally, patients must endure lifelong multiple daily insulin injections and frequent fluctuations in blood glucose (BG) concentrations [6,7,8]. Given the increasing global incidence of T1DM [9], alternative therapeutic options beyond insulin therapy are highly desirable.

Leptin, a hormone secreted by adipocytes, regulates energy balance by acting on neurons within the central nervous system [10]. Notably, intracerebroventricular leptin administration has been shown to normalize glucose metabolism in rodent models of T1DM in the absence of insulin [11,12]. Leptin improves glucose metabolism by acting on hypothalamic arcuate neurons, suppressing hepatic gluconeogenesis, and promoting glucose uptake in muscle and brown adipose tissue via autonomic nervous system activation [13,14]. Compared with insulin administration, treatment with leptin is associated with a lower risk of hypoglycemia, directly inhibits fat synthesis [14,15,16,17] and is an effective treatment for ketoacidosis, a life-threatening complication of T1DM [18]. However, peripheral leptin administration has limited effects on glucose metabolism, as demonstrated in both rodent and human studies [12,18,19]. To overcome this limitation, we previously reported that the combination of leptin administered peripherally with a protein tyrosine phosphatase 1B (PTP1B) inhibitor―an enhancer of leptin receptor signaling―improved glucose metabolism in a mouse model of IDDM in the absence of insulin [20].

PTP1B, the first protein tyrosine phosphatase identified from the human placenta [21], is a 50 kDa non-receptor tyrosine phosphatase localized to the endoplasmic reticulum and is widely expressed throughout the body [22,23]. It inhibits both insulin and leptin receptor signaling by dephosphorylating tyrosine residues [24]. For example, leptin administration during PTP1B deficiency enhances signal transducer and activator of transcription 3 (STAT3) phosphorylation via Janus activating kinase 2 dephosphorylation [25,26]. Notably, PTP1B deficiency in hypothalamic neurons improves glucose metabolism independently of its effects on energy balance [27,28,29]. For IDDM, the glucose-lowering effect of leptin combined with a PTP1B inhibitor has been attributed to PTP1B inhibition in pro-opiomelanocortin neurons in the hypothalamic arcuate nucleus (ARC) [20]. Although PTP1B inhibitors have demonstrated efficacy in phase II clinical trials for the treatment of obesity in patients with type 2 diabetes [30], they have not yet been approved for clinical use.

Interestingly, Kanoski et al. reported that subcutaneous administration of the glucagon-like peptide-1 receptor agonist, liraglutide, downregulated the expression of hypothalamic PTP1B [31]. Glucagon-like peptide-1 receptor agonists, primarily used as a treatment for type 2 diabetes, improve glucose metabolism by stimulating pancreatic insulin secretion, inhibiting glucagon release, increasing glucose uptake in muscle and adipose tissue, and suppressing hepatic gluconeogenesis [32].

Given the potential of liraglutide to reduce the expression of hypothalamic PTP1B, we hypothesized that the combination of leptin and liraglutide would improve glucose metabolism in streptozotocin (STZ)-induced insulin-deficient mice. Our findings demonstrated that peripheral co-administration of leptin and liraglutide significantly improved glucose metabolism in mice with IDDM without the need for insulin administration.

## 2. Results

### 2.1. The Peripheral Co-Administration of Leptin and Liraglutide Improved Hyperglycemia in Mice with IDDM

**Figure 1:**
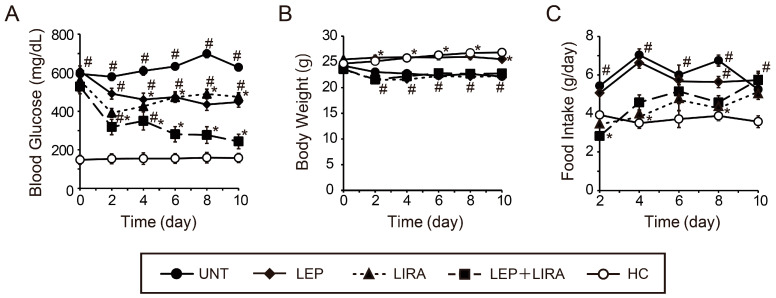
Effects of the administration of leptin and/or liraglutide, a glucagon-like peptide-1 receptor agonist, on blood glucose (BG) and energy metabolism in mice. (**A**) Non-fasting BG. (**B**) Body weight. (**C**) Food intake. Four groups of mice had insulin-dependent diabetes mellitus (IDDM): untreated (UNT), treated with leptin alone (LEP), treated with liraglutide alone (LIRA), co-administered leptin and liraglutide (LEP+LIRA); one group of mice (without IDDM) served as a healthy control (HC). * *p* < 0.05 vs. UNT. ^#^ *p* < 0.05 vs. HC. Significant differences between groups are further detailed in Table S1.

BG levels among the groups of mice with IDDM that were treated with leptin alone (LEP group) were significantly lower than those untreated (UNT group) from days 4 to 10, and BG in both of those groups was significantly higher than in the healthy controls (HC, the only group without IDDM) from days 0 to 10 (Figure 1A). Mice treated with liraglutide alone (LIRA group) had significantly lower BG than the UNT group from days 2 to 10, although BG levels were significantly higher than in the HC group (Figure 1A). There were no significant differences in BG levels between the LEP and LIRA groups (Figure 1A). The BG levels in the group co-administered leptin and liraglutide (LEP+LIRA group) were not only significantly lower than those in the UNT group from days 2 to 10, but BG levels were also significantly lower than those in the LEP and LIRA groups from days 6 to 10 (Figure 1A). Moreover, no significant differences in BG were observed between the LEP+LIRA and HC groups from days 6 to 10 (Figure 1A).

Body weight (BW) in the UNT, LIRA, and LEP+LIRA groups was significantly lower than that in the HC group from days 2 to 10. In contrast, no significant differences were observed between the LEP and HC groups (Figure 1B). The amount of food consumed in the UNT and LEP groups was significantly higher than that in the HCs from days 2 to 10, and no significant differences were observed between both the LIRA and LEP+LIRA groups and HCs from days 2 to 8. However, the amount of food consumed in the LIRA and LEP+LIRA groups was significantly higher than that in the HC group on day 10 (Figure 1C).

On day 10 of the experiment, BG levels in the LEP+LIRA group were similar to those in the HC group; however, their BW was lower, and their food intake was higher compared with HC mice.

### 2.2. Peripheral Co-Administration of Leptin and Liraglutide Improved Glucose Tolerance in Mice with IDDM

**Figure 2:**
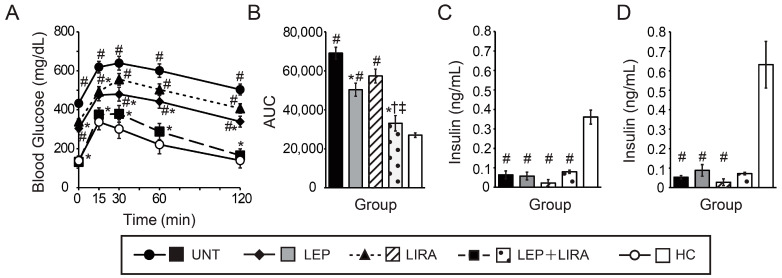
Effects of the administration of leptin and/or liraglutide on glucose tolerance in mice with IDDM. (**A**) Blood glucose levels during the intraperitoneal glucose tolerance test. (**B**) Total changes in blood glucose levels over time are expressed as the area under the curve. (**C**) Serum insulin levels before glucose injection. (**D**) Serum insulin levels 30 min after glucose injection. Four groups of mice had IDDM: untreated (UNT), treated with leptin (LEP), treated with liraglutide (LIRA), and co-administered leptin and liraglutide (LEP+LIRA); one group of mice (without IDDM) served as a healthy control (HC). * *p* < 0.05 vs. UNT. ^†^ *p* < 0.05 vs. LEP. ^‡^ *p* < 0.05 vs. LIRA. ^#^ *p* < 0.05 vs. HC. Significant differences between groups are further detailed in Table S2.

On day 10, all groups underwent a glucose tolerance test (GTT; glucose 2 g/kg BW) to evaluate glucose metabolism. The GTT was performed after fasting for 2 h, based on previous reports [17,33,34], because mice with IDDM treated with leptin develop severe hypoglycemia after prolonged fasting. BG levels in the UNT group were significantly higher than those in the HC group at all time points (Figure 2A). The BG levels in the LEP group were significantly lower than those in the UNT at all time points (Figure 2A). In the LIRA group, BG levels at 0 and 15 min were significantly lower than those in the UNT group, although no significant differences were observed at 30, 60, and 120 min (Figure 2A). In the LEP group, BG levels at 0, 30, 60, and 120 min remained significantly higher than for HC mice, yet no differences were observed at 15 min between LEP and HC mice (Figure 2A). BG levels in the LIRA group were significantly higher than those of HC mice at all time points (Figure 2A).

BG levels in the LEP+LIRA group were significantly lower than those for UNT mice at all time points (Figure 2A). Additionally, BG levels at 0 and 120 min in the LEP+LIRA group were significantly lower than those in the LEP group, and no significant differences were observed at 15, 30, or 60 min between the LEP and LEP+LIRA groups (Figure 2A). Compared with the LIRA group, the LEP+LIRA group had significantly lower BG levels at all time points except at the 15-min mark (Figure 2A). There were no significant differences in BG levels between the LEP+LIRA and HC groups at any time point (Figure 2A). Combination therapy with leptin and liraglutide in mice with IDDM reduced BG levels to those observed in the healthy mice and showed greater efficacy than treatment with leptin or liraglutide alone.

The area under the curve (AUC) derived from the GTT for the UNT group was significantly higher than for the LEP, LEP+LIRA, and HC groups, and no significant differences were observed between the UNT and LIRA groups (Figure 2B). The AUC for LEP+LIRA mice was significantly lower than that for the LEP and LIRA groups, and no significant difference was observed between the LEP+LIRA and HC groups (Figure 2B). Serum insulin levels were measured before (0 min) and 30 min after glucose administration during the GTT. Insulin levels in the UNT, LEP, LIRA, and LEP+LIRA groups were below measurement sensitivity (<0.1 ng/mL) and significantly lower than those in the HC group at both time points (Figure 2C,D).

### 2.3. Liraglutide Treatment Failed to Restore Pancreatic β Cell Area in Mice with IDDM

**Figure 3:**
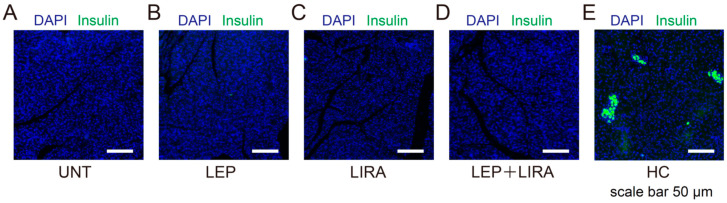
Effects of the administration of leptin and/or liraglutide on pancreatic β cells in mice. Panels show representative stained pancreatic sections of mice with IDDM that were (**A**) untreated (UNT), (**B**) treated with leptin (LEP), (**C**) treated with liraglutide (LIRA), (**D**) co-administered leptin and liraglutide (LEP+LIRA). (**E**) Healthy control (HC) without IDMM. Pancreatic sections were stained with antibodies against 4′,6-diamidino-2-phenylindole (DAPI; blue) and insulin (green).

Immunostaining of the excised pancreas using an insulin antibody revealed that the β-cell area, present in the HC mice (Figure 3E), was undetectable in UNT mice (Figure 3A). It was also undetectable in the LEP, LIRA and LEP+LIRA groups (Figure 3B–D). These findings confirm that β-cell destruction induced by high-dose STZ cannot be rescued via leptin, liraglutide, or their combination and is consistent with the results shown in Figure 2C,D.

### 2.4. Liraglutide Treatment Resulted in Decreased PTP1B Expression in the Hypothalamic Arcuate Nucleus and Enhanced Leptin Receptor Signaling in Mice with IDDM

**Figure 4:**
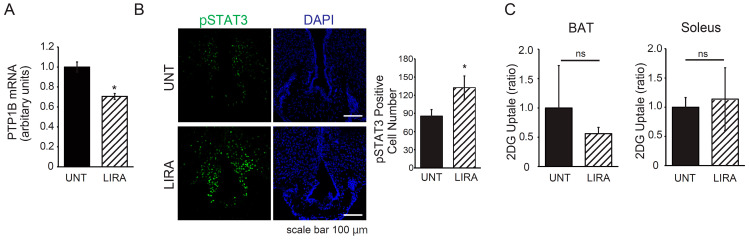
Effects of the administration of liraglutide on PTP1B expression in the arcuate nucleus (ARC) and 2DG uptake in the peripheral tissues of mice with IDDM (**A**) PTP1B mRNA expression in the ARC, (**B**) STAT3 phosphorylation in the ARC (representative samples), and (**C**) 2DG uptake in brown adipose tissue (BAT), and soleus muscle. Untreated [UNT] mice shown in panels for contrast). * *p* < 0.05 vs. UNT mice. ns = not significant. Significant differences between the groups are further detailed in Table S3.

In mice with IDDM, leptin treatment alone had a limited effect on improving hyperglycemia, but co-administration of leptin and liraglutide improved glucose metabolism to levels comparable to those of healthy mice (Figure 1A and Figure 2A). To investigate the mechanism underlying this effect, we examined changes in PTP1B mRNA expression in the arcuate nucleus (ARC) of mice with IDDM who were treated with liraglutide. We confirmed that liraglutide decreased PTP1B mRNA expression in the ARC of these mice (Figure 4A). Consistent with these results, intraperitoneal administration of leptin in mice with IDDM significantly enhanced STAT3 phosphorylation in the ARC in the LIRA group compared with the UNT group (Figure 4B). In addition, the uptake of 2DG in brown adipose tissue (BAT) and the soleus muscle of LIRA mice showed no significant difference compared with the UNT group (Figure 4C). These findings suggest that liraglutide alone does not enhance glucose uptake via a direct action on BAT or muscle. Instead, it potentiated the effect of leptin administration on glucose metabolism by enhancing leptin receptor signaling in the ARC.

### 2.5. Leptin and Liraglutide Improved Hypercorticosteronemia, but Only Leptin Improved Hyperketonemia and Hyperglucagonemia in Mice with IDDM

**Figure 5:**
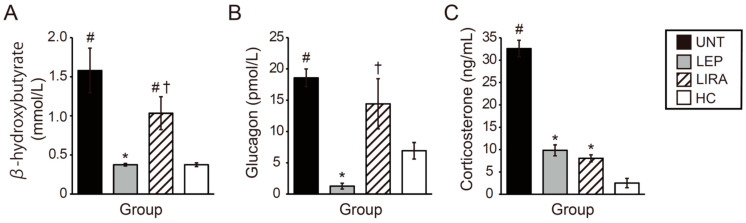
Effects of the administration of leptin or liraglutide on plasma levels of β-hydroxybutyrate (BH) and serum levels of glucagon and corticosterone in mice. (**A**) BH levels. (**B**) Glucagon levels. (**C**) Corticosterone levels. Groups of mice with IDDM included those that were untreated (UNT), treated with leptin (LEP), and treated with liraglutide (LIRA); one group of mice (without IDDM) served as a healthy control (HC). * *p* < 0.05 vs. UNT. ^†^ *p* < 0.05 vs. leptin. ^#^ *p* < 0.05 vs. HC. Significant differences between the groups are further detailed in Table S4.

Plasma levels of β-hydroxybutyrate (BH) in the UNT and LIRA groups were significantly higher than those in the LEP and HC groups on day 10. However, no significant differences were observed in plasma BH between the UNT and LIRA groups and between the LEP and HC groups (Figure 5A).

Serum glucagon levels in the UNT and LIRA groups were significantly higher than those in the HC group on day 10, and no significant differences were observed between them (Figure 5B). In contrast, serum glucagon levels in the LEP group were significantly lower than those in the UNT group (Figure 5B).

Serum corticosterone levels in the UNT group were significantly higher than those in the LEP, LIRA and HC groups on day 10, and no significant differences were observed among the LEP, LIRA, and HC groups (Figure 5C). Thus, in IDDM mice, both leptin and liraglutide improved hypercorticosteronemia, but only leptin improved hyperketonemia and hyperglucagonemia.

## 3. Discussion

This study evaluated the effects of the peripheral co-administration of leptin and liraglutide on glucose metabolism in a mouse model of IDDM. Our results revealed that, together, leptin and liraglutide improved glucose metabolism in mice with STZ-induced IDDM to levels comparable with those of healthy mice without the use of insulin. These results suggest that combination therapy with leptin and liraglutide could be a potential treatment option for IDDM.

The glucose-lowering effects of the co-administration of leptin and liraglutide were not mediated by insulin receptor signaling, as insulin concentrations in the mice with IDDM were undetectable at 0 and 30 min during a GTT. Additionally, in the UNT group, food intake was significantly higher, and BW was significantly lower compared with the HC group, as was reported previously [20,35]. However, on day 10, no significant differences in food intake or BW were observed between the LEP+LIRA and UNT groups. Collectively, our results suggest that the improvement in glucose metabolism observed in mice with IDDM co-administered leptin and liraglutide was due to enhanced leptin receptor signaling in the hypothalamus, independent of insulin receptor signaling, food intake, or BW changes.

Central administration of leptin to a mouse model of IDDM has been shown to improve glucose metabolism to levels comparable to those of normal mice, even in the absence of insulin [11,12]. Regarding the mechanism by which leptin ameliorates hyperglycemia, previous studies have reported that leptin acts on hypothalamic neurons to inhibit hepatic gluconeogenesis and to promote glucose uptake in BAT and muscle via autonomic nervous system pathways [13,14,20,36,37]. Consistent with these findings, peripheral administration of leptin has also been shown to alleviate hyperketonemia [35,38], hyperglucagonemia [20,39], and hypercorticosteronemia [20,39] in IDDM rodent models. However, multiple independent studies have indicated that the hypoglycemic effect of peripheral leptin administration is limited, posing challenges for clinical application [12,18,19]. This aligns with the results of the present study. In this context, we previously reported that the co-administration of leptin and a PTP1B inhibitor, which enhanced leptin receptor signaling, improved glucose metabolism in a mouse model of IDDM [20]. Furthermore, we demonstrated that the enhancement of leptin signaling via PTP1B inhibition in the hypothalamic ARC played a critical role in that improvement [20].

In the present study, we confirmed that the peripheral administration of liraglutide reduced the expression of PTP1B in the hypothalamic ARC and simultaneously enhanced leptin signaling in the same region, consistent with previous reports [31]. Our data showed that liraglutide alone did not enhance glucose uptake in peripheral tissues such as BAT and muscle, nor did it reduce hyperketonemia in mice with IDDM. Therefore, the enhancement of leptin signaling in the hypothalamic ARC induced by liraglutide can be considered a possible mechanism underlying the improvement in glucose metabolism observed with the combination treatment of leptin and liraglutide in mice with IDDM. Furthermore, although we demonstrated that liraglutide reduced PTP1B expression in the hypothalamic ARC, the precise molecular mechanism remains to be fully elucidated. It is possible that by activating GLP-1 receptors on ARC neurons, liraglutide indirectly modulates intracellular signaling pathways, such as the cyclic AMP/protein kinase A pathway and phosphatidylinositol 3-kinase pathway [40], ultimately leading to downregulation of PTP1B transcription. In addition, while we observed enhanced leptin receptor signaling in the ARC, it remains unclear which specific neuronal populations are predominantly involved. Given the established role of pro-opiomelanocortin neurons in leptin-mediated glucose regulation in mice with IDDM [20], it is plausible that liraglutide and leptin co-treatment synergistically enhanced leptin receptor signaling in POMC neurons, thereby contributing to improved glucose homeostasis. Future studies utilizing neuron subtype-specific approaches will be necessary to clarify the precise cellular targets and signaling cascades underlying these effects.

Glucagon-like peptide-1 receptor agonists, used for the treatment of type 2 diabetes, have been reported to exert two beneficial effects on pancreatic islet hormone secretion: the promotion of insulin secretion and the suppression of glucagon secretion [41]. These interactions are believed to be key contributors to glucagon-like peptide-1 receptor agonist-induced hypoglycemia [42]. However, in the mouse model of IDDM used in this study, generated using high doses of STZ, administration of liraglutide neither stimulated insulin secretion nor inhibited glucagon secretion. The improvement in glucose metabolism observed in LIRA mice, compared with the UNT group, may be attributed to several factors, such as a reduction of food intake until day 8 of the experiment and the alleviation of hypercorticosteronemia. The role of glucagon-like peptide-1 receptor agonists in type 1 diabetes has been investigated using exenatide and liraglutide, which have been reported to reduce insulin requirements, promote weight loss, and improve HbA1c levels [43]. However, these effects are most pronounced in patients with detectable C-peptide levels or those who are overweight, among other factors [43]. Given that overweight patients with type 1 diabetes are already hyperleptinemic [44,45,46], the findings of this study suggest that the enhancement of leptin signaling in the hypothalamic ARC using liraglutide treatment may contribute to weight loss and improved glucose metabolism.

An important result of this study is that liraglutide not only suppressed the expression of PTP1B in mice with impaired glucose metabolism, but it aligns with the suppressed expression of PTP1B in the hypothalamus of normal rats on a standard diet [31]. Since PTP1B inhibitors are known to improve glucose and energy metabolism by enhancing leptin and insulin receptor signaling [24,30], further investigation is warranted to elucidate the molecular mechanisms by which liraglutide suppresses PTP1B expression.

Insulin administration remains the only first-line treatment for patients with IDDM. The need for improved treatments of IDDM remains unmet due to the potential drawbacks of insulin therapy, including an increased risk of life-threatening hypoglycemia and weight gain, which are associated with insulin resistance and cardiovascular events [47]. Additionally, long-term insulin use can lead to side effects such as the development of insulin antibodies and insulin allergy [48,49]. Despite continuous advancements in insulin delivery technologies and glucose monitoring systems, fewer than one-third of patients with IDDM achieve optimal glycemic management [50]. Based on the data obtained in this study, the combined administration of leptin and liraglutide may offer benefits for patients with IDDM who struggle with glycemic management despite insulin therapy.

Several limitations of this study should be acknowledged. First, we did not explore the optimal doses of leptin and liraglutide, nor the potential interactions of combination therapy with residual insulin secretion in models with partial β-cell preservation. Second, the optimal body mass index range for the effectiveness of this treatment remains unclear. Third, although the STZ-induced model of IDDM replicates key features of human T1DM, it does not fully recapitulate the complex immunological mechanisms underlying T1DM pathogenesis. Future studies addressing these issues will be necessary to validate and extend our findings.

## 4. Materials and Methods

### 4.1. Animal Model and Experimental Design

11-week-old male wild-type (C57BL/6J) mice purchased from Japan SLC, Inc. (Shizuoka, Japan) were housed under a 12-h light/12-h dark cycle in a temperature-controlled barrier facility. Mice were fed a standard diet ad libitum and had free access to water. All animal procedures were approved by the Animal Care and Use Committee of Nagoya University Graduate School of Medicine and performed in accordance with the institutional guidelines that conform to the National Institutes of Health animal care guidelines.

At 12–13 weeks of age, IDDM was induced via intraperitoneal injection of STZ (150 mg/kg; Sigma-Aldrich, St Louis, MO, USA). STZ was dissolved in 0.1 M sodium citrate buffer (1.47 g sodium citrate in 50 mL, pH 8.0) immediately before injection. Non-fasting BG levels were measured 2 and 7 days after each STZ injection. One week later, BG levels exceeded 400 mg/dL, and serum levels of insulin were less than 0.1 ng/mL in the IDDM mice. STZ treatment was used to successfully induce IDDM in mice with an average BG level ≥ 400 mg/dL, and those mice were selected for subsequent drug administration and experiments. In this study, mice with IDDM were defined as those rendered completely insulin deficient by high-dose STZ administration. This experimental model is pathophysiologically distinct from human type 1 diabetes mellitus (T1DM), which is caused primarily by autoimmune destruction of pancreatic β-cells.

Seven days after the administration of STZ, mice with IDDM were divided into four groups as follows: untreated (UNT; vehicle), treated with leptin (LEP), treated with the glucagon-like peptide-1 receptor agonists liraglutide (LIRA), and treated with leptin and liraglutide (LEP+LIRA). One group of mice without IDDM was used as a healthy control (HC; vehicle). All mice were subcutaneously implanted with osmotic pumps in the mid-scapular region (Alzet model 1002; Durect Corporation, Cupertino, CA, USA). Each pump chronically delivered either saline (UNT, LIRA and HC) or recombinant mouse leptin (LEP and LEP+LIRA) for 10 days. The dosages of liraglutide and leptin (National Hormone and Peptide Program, Torrance, CA, USA) were set at 1000 µg/kg/day and 20 µg/day, respectively, based on previous reports [20,51,52,53]. Liraglutide was administered with a subcutaneous injection once daily. For all five groups, BG, BW, and the amount of food consumed were monitored every 2 days.

### 4.2. In Vivo Glucose Homeostasis

BG levels were measured in blood drawn from the tail vein using Glutest Mint^®^ tests (Sanwa Kagaku Kenkyusyo, Nagoya, Japan) to measure BG levels up to 1000 mg/dL. An intraperitoneal GTT was performed in each mouse on day 10. For GTTs, the mice were intraperitoneally injected with 2.0 g/kg glucose after fasting for 2-h, and BG was measured at 0, 15, 30, 60, and 120 min thereafter. Blood samples were obtained from the tail vein at 0 and 30 min to evaluate serum insulin levels, and the area under the curve (AUC) for glucose was then calculated to quantify the total increase in BG during the GTT. Beta-hydroxybutyrate, a type of ketone body, was measured using a FreeStyle Libre Reader^®^ meter (Abbott Diabetes Care, Alameda, CA, USA) and FreeStyle Optium β-Ketone test strips. Serum insulin content was measured using an Ultrasensitive Mouse Insulin enzyme-linked immunosorbent assay (Morinaga, Tokyo, Japan).

### 4.3. Blood and Tissue Samples

Prior to euthanasia, the submandibular vein was lanced for blood collection. Plasma was prepared by centrifugation (1500× *g*, 10 min, 4 °C) and stored at −80 °C. Mice were euthanized by cervical dislocation, except for those subjected to immunohistochemistry. For immunohistochemical evaluation, mice were anesthetized and perfused as described below. Their organs were removed, weighed, dissected, and snap-frozen in liquid nitrogen and stored at −80 °C until required.

### 4.4. Measurement of Serum Glucagon and Corticosterone Levels

Submandibular blood was collected, and the serum was separated by centrifugation at 3500× *g* for 10 min. Glucagon and corticosterone levels were measured using an enzyme-linked immunosorbent assay (Funakoshi, Tokyo, Japan).

### 4.5. Measurement of 2-Deoxyglucose Uptake

To measure 2-deoxyglucose uptake (2DG) in peripheral tissues, we assessed the UNT and LIRA groups of mice (see Section 4.1). 2DG was administered at a concentration of 5 μM dissolved in 0.1 mL of 0.9% saline (NaCl) per mouse. On day 10, after the administration of saline and liraglutide, respectively, both groups were fasted for 4 h. Subsequently, 2DG (5 μM) was administered intraperitoneally, and the mice were euthanized 20 min later to collect BAT and soleus muscle samples. For measurement of 2DG content, tissue samples (10 mg for BAT and muscle) were homogenized in 500 μL of 10 mM Tris-HCl (pH 8.1), heated to 95 °C for 10 min, and then centrifuged at 15,000 rpm for 10 min at 4 °C. The supernatants were diluted (10×) with 10 mM Tris-HCl (pH 8.1) and assayed for 2DG content according to the 2DG Uptake Measurement Kit (Funakoshi, Tokyo, Japan).

### 4.6. Immunohistochemistry

STAT3 phosphorylation was detected using immunohistochemistry. For detection, mice were fasted overnight (12 h) and injected intraperitoneally with mouse recombinant leptin (1 μg/g BW) as described previously [26,28]. Forty-five minutes after leptin injection, mice were anesthetized by intraperitoneal administration of a mixture of medetomidine (0.3 mg/kg), midazolam (4 mg/kg), and butorphanol (5 mg/kg), followed by transcardial perfusion with a cold fixative containing 4% paraformaldehyde in phosphate-buffered saline (PBS) at pH 7.4. After fixation, the brains were removed and immersed in the same fixative for 2 h at 4 °C. The brains were transferred into PBS containing 10–20% sucrose at 4 °C for cryoprotection. Thereafter, the brains were embedded in the Tissue-Tek O.C.T. compound (Sakura Finetek, Tokyo, Japan) and stored at −80 °C prior to sectioning. Sections (20-μm thick) of the brain were sliced on a cryostat at −20 °C, thawed and mounted on Superfrost Plus microscope slides (Matsunami, Osaka, Japan). The sections were stored at −80 °C until immunohistochemistry. Immunohistochemistry of STAT3 phosphorylation was performed as described previously [26,28]. Sections were washed with 1 × PBS prior to and between successive blocking steps with 1.0% H_2_O_2_ /1.0% NaOH in H_2_O for 20 min, 0.3% glycine for 10 min, 0.03% sodium dodecyl sulfate for 10 min, and finally 0.2% sodium azide/3% normal goat serum /0.25% Triton X-100 in PBS for 1 h at room temperature. The sections were then incubated with anti-STAT3 phosphorylation antibody (1:100 dilution: Cell Signaling, Danvers, MA, USA) and diluted in azide blocking solution overnight at 4 °C. Sections were washed in 1 × PBS and incubated in sodium azide-free blocking solution containing Alexa Fluor 488-conjugated anti-rabbit IgG secondary antibody (1:500: Invitrogen, Carlsbad, CA, USA) for 1 h at room temperature. After washing in 1 × PBS, sections were placed on slides, air dried, mounted using VECTASHIELD (Vector Labs, Newark, CA, USA) and coverslipped. All sections that underwent fluorescent staining were examined using a confocal laser microscope (TiEA1R; Nikon Instech, Tokyo, Japan) and viewed using NIS-Elements AR software (version 5.11.01; Nikon Instech, Tokyo, Japan). Cells labeled for phospho-STAT3 were counted bilaterally in a blinded fashion. Between 4 and 6 mice in each group were analyzed by calculating the mean value of 3 or 4 serial sections per mouse. The sections included the arcuate nucleus, −1.70 mm to −1.82 mm from bregma based on coordinates in the brain atlas [54]. All fluorescently stained sections were examined using a confocal laser microscope (TiEA1R; Nikon Instech, Tokyo, Japan) and viewed using NIS-Elements software (RRID:SCR_014329; TiEA1R; Nikon Instech, Tokyo, Japan).

Pancreatic tissues were fixed using 4% paraformaldehyde at 4 °C and subsequently embedded in paraffin. Serial sections 4-μm thick were obtained from each paraffin block at 200-μm intervals. Five sections per mouse were randomly chosen for further analysis. After deparaffinization, serial sections were treated with a rabbit anti-insulin recombinant monoclonal antibody (1:5000; [EPR17359] catalog # ab181547; Abcam, Cambridge, UK) for 24 h at 4 °C to examine the proliferation of insulin-positive cells. Subsequently, the sections were treated with Alexa Fluor 488-conjugated goat anti-rabbit IgG secondary antibody (1:500; catalog # A11034; Invitrogen, Carlsbad, CA, USA) for 1.5 h at room temperature. All sections stained with fluorescent dyes were examined using a BZ9000 fluorescent microscope system (Keyence, Osaka, Japan).

### 4.7. Gene Expression Analysis

Total RNA was extracted from samples using TRIzol (Invitrogen, Carlsbad, CA, USA) and a RNeasy kit (Qiagen, Hilden, Germany). Copy DNA was synthesized from 100 ng total RNA using a ReverTra Ace qPCR RT Kit (Toyobo, Osaka, Japan). Quantitative reverse transcriptase (qRT)–PCR reactions were performed using Brilliant III Ultra-Fast SYBR Green QPCR Master Mix (Agilent Technologies, Santa Clara, CA, USA), and samples were run using a CFX Connect Real Time-PCR Detection System (Bio-Rad, Hercules, CA, USA). The relative mRNA levels of protein tyrosine phosphatase non-receptor type 1 (*Ptpn1*) in ARC explants were assessed by qRT–PCR using glyceraldehyde 3-phosphate dehydrogenase (*Gapdh*) as an internal control [*Ptpn1* Forward: 5′-GCGCTTCTCCTACCTGGCTGTCAT-3′; Reverse: 5′-ACGTGCTCGGGTGGAAGGTCTA-3, *Gapdh* Forward: 5′-AGGTCGGTGTGAACGGATTTG-3′; Reverse: 5′-TGTAGACCATGTAGTTGAGGTCA-3]. The qRT–PCR reactions were performed, and relative mRNA expression levels were calculated using a comparative cycle threshold or Ct method as described previously [26].

### 4.8. Statistical Analysis

The statistical significance of the differences between groups was analyzed using either unpaired *t*-tests, Kruskal-Wallis tests, one-way ANOVA, or two-way ANOVA with repeated measures followed by Bonferroni’s test as appropriate. Results are expressed as mean ± standard error of the mean (SEM), and differences were considered statistically significant at *p* < 0.05. Information on the number of samples per experiment and the statistical analyses performed for intergroup comparisons is presented in Tables S1–S3.

## 5. Conclusions

Peripheral combination treatment with leptin and liraglutide ameliorated glucose metabolism in mice with STZ-induced IDDM without the use of insulin.

## Data Availability

The data presented are available upon request from the corresponding author.

## Supplementary Materials

The supporting information can be downloaded at: https://www.mdpi.com/article/10.3390/ijms26104595/s1.

## Abbreviations

The following abbreviations are used in this manuscript:

IDDM	Insulin-dependent diabetes mellitus
BG	Blood glucose
GTT	Glucose tolerance test
T1DM	Type 1 diabetes mellitus
PTP1B	Protein tyrosine phosphatase 1B
STAT3	Signal transducer and activator of transcription 3
ARC	Arcuate nucleus
STZ	Streptozotocin
BW	Body weight
AUC	Area under the curve
2DG	2-deoxyglucose
BAT	Brown adipose tissue
BH	β-hydroxybutyrate
