# Supplementary material for: Effects of Combination Treatment with Leptin and Liraglutide on Glucose Metabolism in Insulin-Dependent Diabetic Mice
Source: Int J Mol Sci. 2025 May 11;26(10):4595. doi: 10.3390/ijms26104595 (PMC12111290; doi:10.3390/ijms26104595)
Supplement: Supplementary file 1 [file ijms-26-04595-s001.zip › Table S1.pdf]

Table S1. The details of statistics used in this study.

Figure	Panel	Number of sample	Test used	F/t/p value and degrees of freedom (df)	Post hoc test	Significance
1A	Blood Glucose (BG)	UNT=35<br>LEP=16<br>LIRA=24<br>LEP+LIRA =8<br>HC=20	Two-way ANOVA assessed by repeated measures	Time: F(5, 490) = 15.189, p < 0.001<br>Group: F(4, 98) =103.278, p < 0.001<br>Interaction: F(20, 490) = 6.936, p < 0.001	Bonferroni	day0: A vs E; B vs E; C vs E; D vs E<br>day2: A vs C; A vs D; A vs E; B vs D; B vs E; C vs E; D vs E<br>day4: A vs B; A vs C; A vs D; A vs E; B vs E; C vs E; D vs E<br>day6: A vs B; A vs C; A vs D; A vs E; B vs D; B vs E; C vs D; C vs E<br>day8: A vs B; A vs C; A vs D; A vs E; B vs D; B vs E; C vs D; C vs E<br>day10: A vs B; A vs C; A vs D; A vs E; B vs D; B vs E; C vs D; C vs E
1B	Body Weight (BW)	UNT=32<br>LEP=16<br>LIRA=23<br>LEP+LIRA=8<br>HC=19	Two-way ANOVA assessed by repeated measures	Time: F(5, 465) = 10.148, p < 0.001<br>Group: F(4, 93) =18.055, p < 0.001<br>Interaction: F(20, 465) = 13.077, p < 0.001	Bonferroni	day0: B vs C<br>day2: A vs B; A vs C; A vs E; B vs C; B vs D; C vs E; D vs E<br>day4: A vs B; A vs E; B vs C; B vs D; C vs E; D vs E<br>day6: A vs B; A vs E; B vs C; B vs D; C vs E; D vs E<br>day8: A vs B; A vs E; B vs C; B vs D; C vs E; D vs E<br>day10: A vs B; A vs E; B vs C; C vs E; D vs E
1C	Food Intake	UNT=6<br>LEP=6<br>LIRA=5<br>LEP+LIRA=4<br>HC=8	Two-way ANOVA assessed by repeated measures	Time: F(4, 96) = 11.944, p < 0.001<br>Group: F(4, 24) =13.865, p < 0.001<br>Interaction: F(16, 96) = 5.906, p < 0.001	Bonferroni	day2: A vs C; A vs D; A vs E; B vs C; B vs D<br>day4: A vs C; A vs E; B vs C; B vs E<br>day6: A vs E; B vs E; D vs E<br>day8: A vs C; A vs D; A vs E; B vs E<br>day10: A vs E; B vs E; C vs E; D vs E

UNT=A; LEP=B; LIRA=C; LEP+LIRA=D; HC=E.
