# Supplementary material for: Effects of Combination Treatment with Leptin and Liraglutide on Glucose Metabolism in Insulin-Dependent Diabetic Mice
Source: Int J Mol Sci. 2025 May 11;26(10):4595. doi: 10.3390/ijms26104595 (PMC12111290; doi:10.3390/ijms26104595)
Supplement: Supplementary file 1 [file ijms-26-04595-s001.zip › Table S2.pdf]

Table S2. The details of statistics used in this study.

Figure	Panel	Number of sample	Test used	F/t/p value and degrees of freedom (df)	Post hoc test	Significance
2A	GTT<br>Blood Glucose	UNT=13<br>LEP=13<br>LIRA=18<br>LEP+LIRA=9<br>HC=7	Two-way ANOVA assessed by repeated measures	Time: $F(4, 220) = 104.719$, $p < 0.001$<br>Group: $F(4, 55) = 23.006$, $p < 0.001$<br>Interaction: $F(16, 220) = 1.591$, $p < 0.001$	Bonferroni	0min: A vs B; A vs C; A vs D; A vs E; B vs D; B vs E; C vs D; C vs E<br>15min: A vs B; A vs C; A vs D; A vs E; C vs E<br>30min: A vs B; A vs D; A vs E; B vs E; C vs D; C vs E<br>60min: A vs B; A vs D; A vs E; B vs E; C vs D; C vs E<br>120min: A vs B; A vs D; A vs E; B vs D; B vs E; C vs D; C vs E
2B	GTT<br>AUC	UNT=13<br>LEP=13<br>LIRA=18<br>LEP+LIRA=9<br>HC=7	Kruskal-Wallis	Group: $F(4, 59) = 19.807$, $p < 0.001$	Bonferroni	A vs B; A vs D; A vs E; B vs D; B vs E; C vs D; C vs E
2C	Insulin<br>0 min	UNT=11<br>LEP=5<br>LIRA=5<br>LEP+LIRA=5<br>HC=4	One-Way ANOVA	Group: $F(4, 29) = 27.778$, $p < 0.001$	Bonferroni	A vs E; B vs E; C vs E; D vs E
2D	Insulin<br>30 min	UNT=14<br>LEP=5<br>LIRA=6<br>LEP+LIRA=6<br>HC=4	One-Way ANOVA	Group: $F(4, 34) = 40.560$, $p < 0.001$	Bonferroni	A vs E; B vs E; C vs E; D vs E

UNT=A; LEP=B; LIRA=C; LEP+LIRA=D; HC=E.
