# Supplementary material for: Effects of Combination Treatment with Leptin and Liraglutide on Glucose Metabolism in Insulin-Dependent Diabetic Mice
Source: Int J Mol Sci. 2025 May 11;26(10):4595. doi: 10.3390/ijms26104595 (PMC12111290; doi:10.3390/ijms26104595)
Supplement: Supplementary file 1 [file ijms-26-04595-s001.zip › Table S3.pdf]

Table S3. The details of statistics used in this study.

Figure	Panel	Number of sample	Test used	F/t/p value and degrees of freedom (df)	Significance
4A	PTP1B mRNA	UNT=8<br>LIRA=6	Unpaired t-test	t=4.609, df=12	UNT vs LIRA, P<0.05
4B	STAT3 phosphorylation	UNT=12<br>LIRA=12	Unpaired t-test	t=-2.087, df=22	UNT vs LIRA, P<0.05
4C	2DG uptake in BAT	UNT=4<br>LIRA=4	Unpaired t-test	t=0.599, df=6	UNT vs LIRA, ns
4C	2DG uptake in soleus muscle	UNT=4<br>LIRA=4	Unpaired t-test	t=-0.246, df=6	UNT vs LIRA, ns
