# Supplementary material for: Effects of Combination Treatment with Leptin and Liraglutide on Glucose Metabolism in Insulin-Dependent Diabetic Mice
Source: Int J Mol Sci. 2025 May 11;26(10):4595. doi: 10.3390/ijms26104595 (PMC12111290; doi:10.3390/ijms26104595)
Supplement: Supplementary file 1 [file ijms-26-04595-s001.zip › Table S4.pdf]

Table S4. The details of statistics used in this study.

Figure	Panel	Number of sample	Test used	F/t/p value and degrees of freedom (df)	Post hoc test	Significance
5A	β-hydroxybutyrate (BH)	UNT=5<br>LEP=8<br>LIRA=6<br>HC=8	One-Way ANOVA	Group: $F(3, 26) = 15.722$, $p < 0.001$	Bonferroni	A vs B; A vs E; B vs C; C vs E
5B	Glucagon levels	UNT=8<br>LEP=3<br>LIRA=5<br>HC=4	One-Way ANOVA	Group: $F(3, 19) = 9.495$, $p < 0.001$	Bonferroni	A vs B; A vs E; B vs C
5C	Corticosterone levels	UNT=8<br>LEP=4<br>LIRA=3<br>HC=3	One-Way ANOVA	Group: $F(3, 17) = 63.066$, $p < 0.001$	Bonferroni	A vs B; A vs C; A vs E

UNT=A; LEP=B; LIRA=C; HC=E.
